# Older Adults’ Experiences With an Online Survey

**DOI:** 10.2196/65684

**Published:** 2025-01-08

**Authors:** Kristie Rebecca Weir, Yehya Maitah, Sarah E Vordenberg

**Affiliations:** 1 Sydney School of Public Health Faculty of Medicine and Health The University of Sydney Sydney Australia; 2 Institute of Primary Health Care (BIHAM) University of Bern Bern Switzerland; 3 Albany Medical College Albany, NY United States; 4 University of Michigan College of Pharmacy Department of Clinical Pharmacy Ann Arbor, MI United States

**Keywords:** older adults, gerontology, geriatric, older, aging, online, internet, survey, questionnaire, research engagement, engagement, study subject, participant, medication decisions

## Abstract

The study explored older adults' perceptions after participating in an online survey about medication decisions, finding that approximately 80% of participants provided positive feedback about the research methodology and their experience.

## Introduction

Older adults are underrepresented in research, often due to age-related biases and stringent exclusion criteria; this limits generalizability and leaves knowledge gaps [1].

The increasing prevalence of online research has the potential to increase older adult participation, given the rising internet use among this demographic [2]. While research has primarily focused on improving recruitment of older adults, an understanding of their experiences of online research is needed to effectively engage older people [3]. While most surveys include some form of piloting, they rarely capture or share participants’ perceptions of the research process. We sought to explore older adults’ perceptions after participating in an online experimental survey, given the potential high accessibility of this type of research.

## Methods

### Overview

We previously reported the main findings from a vignette-based online experiment conducted among adults aged 65 years and older from Australia, the Netherlands, the United Kingdom, and the United States [4]. Participants were recruited for this 15-minute survey through a panel of internet users administered by Qualtrics Research Panels. The study focused on contextual factors influencing how older adults (mean age 71.5, SD 5.1 years) think about deciding to stop a cardiovascular disease medication. In this study, we conducted a content analysis of free-text comments (Textbox 1). The final analysis framework included 17 codes that were subsequently consolidated into 14 themes. Descriptive statistics were used to assess the frequency of each theme.

**Summary of methodology for the content analysis.**

1. A vignette-based online experiment was conducted among adults aged 65 years and older from Australia, the Netherlands, the United Kingdom, and the United States using sampling quotas to ensure balanced representation by country and gender. This study was registered at ClinicalTrials.gov (NCT04676282). At the end of the survey, participants were provided the opportunity to leave “any comments about this study.”
2. A content analysis was conducted among participants who received the vignette about a hypothetical patient, Mrs EF. Participants from the Netherlands were excluded, as the study team for the content analysis was not fluent in Dutch.
3. Study authors read through the comments to inductively generate codes.
4. Two investigators (SEV and YM) independently coded comments, resulting in >80% agreement.
5. All discrepancies in codes were discussed until consensus was reached.

### Ethical Considerations

The online experiment was registered at ClinicalTrials.gov (NCT04676282) and was deemed exempt by the University of Michigan Health Sciences and Behavioral Sciences Institutional Review Board (HUM00183129); by extension, a waiver of consent was granted. All data were collected anonymously. Participants were compensated based on the terms of their panel agreement.

## Results

Participants (N=1789) most frequently did not provide any feedback in the free-text comment field (n=784, 43.8%) or wrote that they had no comments (n=487, 27.2%). Three participants (0.2%) gave unclear statements.

Participants’ comments (n=515, 28.8%) were primarily positive (415/515, 80.6%), such as that the study was interesting (116/515, 22.5%). Themes and representative quotes are reported in Table 1. Participants said it made them think about their health (80/515, 15.5%) and some participants shared further health information about the study topic (48/515, 9.3%). Participants provided feedback on how to improve the study, categorized as question-specific comments (21/515, 4.1%), general suggestions (12/515, 2.3%), or country-specific comments (3/515, 0.6%). Few participants (16/515, 3.1%) provided negative feedback about the survey.

**Table 1:** Older adults’ feedback about an online survey using a hypothetical vignette by theme, with representative quotes among respondents who provided feedback (n=515).

Themes			Representative quotes (participant code)		Participants, n (%)	
**Positive feedback (n=415, 80.6%)**						
	Interesting	“An interesting study” (919) “This was an interesting survey to complete” (2200)		116 (22.5)		
	Thought provoking	“Interesting and insightful. Made me think a little more about how best to manage my health” (1872) “Very good survey to ponder thoughts and beliefs” (481)		80 (15.5)		
	Positive feedback	“Great stuff. Keep up the good work” (2574) “Very good” (764)		67 (13)		
	Thanks for opportunity to participate	“Thanks” (42)		43 (8.3)		
	Positive feedback about survey questions or structure	“Love the format, so easy to see, follow, and understand” (1904) “Very good questions regarding whether or not to stop a medication when you’ve been on it for an extended time” (2026)		35 (6.8)		
	Enjoyed taking survey	“Enjoyed it” (328) “Love doing your studies” (287)		34 (6.6)		
	Unusual study design	“Very different enjoyed the variety” (540) “Unusual but interesting” (1853)		19 (3.7)		
	Interested in results	“Interested in purpose of results” (1863) “Very interesting! Now I need to see the final results” (3481)		15 (2.9)		
	Interested in future studies	“Great study, I’d do more” (4815) “Need more like this” (2516)		6 (1.2)		
**Neutral feedback (n=48, 9.3%)**						
	Shared personal experience	“I am afraid of going to see the dr for worries about my health” (1526) “Thanks; my prescription drugs fall under the Federal Government program where they are funded nearly 100%” (2645)		48 (9.3)		
**Negative feedback or suggestions for improvement (n=52, 10.1%)**						
	Question-specific feedback	“The percentage charts were confusing” (2403) “One question said check all that apply but only one was allowed” (4279)		21 (4.1)		
	Negative feedback	“Boring” (1409) “Too many generalizations” (1102)		16 (3.1)		
	General suggestions for improvement	“Consider adding a progress bar to the survey” (2027) “Good study could be shorter” (2433		12 (2.3)		
	Country-specific feedback	“Some questions are designed for the USA” (1471) “Prescription insurance? In UK if you are over 65 prescriptions are free” (4075)		3 (0.6)		

## Discussion

Among older adults who provided feedback about their experiences completing an online survey, approximately 80% (415/515) of the comments were positive. Our findings signal general acceptability of the methodology, and we have implemented the practical feedback to improve our online surveys. We have become more mindful of the survey length, selected straightforward question types, and have conducted pilot testing in all target countries to ensure that questions are appropriate for all participants.

Our study had several limitations. First, less than one-third of the study participants provided any comments. Second, participants were asked if they had any comments, as opposed to more specific questions about their experience taking the survey. Finally, we acknowledge that we coded a single primary theme per comment given the short statements that were provided.

With an aging population who may spend many years in retirement, participating in research can offer benefits such as reducing social isolation and loneliness, fostering a sense of purpose, and providing mental stimulation, and it may provide monetary incentives [5]. While our online study lacked the social benefit of in-person interaction typical of traditional research, participants reported enjoying the survey; they found it prompted self-reflection on their health, and they expressed interest in the study’s outcomes and future research.

Online research is becoming more prevalent; therefore, it is important to make sure this methodology is inclusive of older adults [5]. More than half of adults older than 65 years use the internet, yet they are the least likely to have a home computer [6]. While some researchers attribute this limited use to age-related functional decline, others argue that the main barriers are negative attitudes such as fear, anxiety, and low motivation—barriers that are modifiable. Anxiety about using the internet and digital technologies often leads to self-imposed limitations and low confidence, with older adults frequently underestimating their knowledge and abilities compared to younger users [6]. This underscores the importance of studies like ours that highlight the positive experiences of older adults in online research.

## References

[1] Markham SC, McNab J, O'Loughlin K, Clemson L (2023). International responses addressing the under-representation of older people in clinical research. Australas J Ageing.

[2] Faverio M Share of those 65 and older who are tech users has grown in the past decade. Pew Research Center.

[3] Remillard ML, Mazor KM, Cutrona SL, Gurwitz JH, Tjia J (2014). Systematic review of the use of online questionnaires of older adults. J Am Geriatr Soc.

[4] Weir KR, Vordenberg SE, Scherer AM, Jansen J, Schoenborn N, Todd A (2024). Exploring different contexts of statin deprescribing: a vignette-based experiment with older adults across four countries. J Gen Intern Med.

[5] Lindeman D (2017). Improving the independence of older adults through technology: directions for public policy. Public Policy Aging Rep.

[6] Vroman KG, Arthanat S, Lysack C (2015). “Who over 65 is online?” Older adults’ dispositions toward information communication technology. Comput Human Behav.

